# A Dual Filtration-Based Multiplex PCR Method for Simultaneous Detection of Viable *Escherichia coli* O157:H7, *Listeria monocytogenes*, and *Staphylococcus aureus* on Fresh-Cut Cantaloupe

**DOI:** 10.1371/journal.pone.0166874

**Published:** 2016-12-01

**Authors:** Ke Feng, Wenzhong Hu, Aili Jiang, Sarengaowa, Yongping Xu, Yu Zou, Liu Yang, Xin Wang

**Affiliations:** 1 School of Life Science and Biotechnology, Dalian University of Technology, Dalian, China; 2 College of Life Science, Dalian Nationalities University, Dalian, China; Agricultural University of Athens, GREECE

## Abstract

Fresh-cut cantaloupe is particularly susceptible to contamination with pathogenic bacteria, such as *Escherichia coli* O157:H7, *Listeria monocytogenes*, and *Staphylococcus aureus*. Therefore, development of rapid, yet accurate detection techniques is necessary to ensure food safety. In this study, a multiplex PCR system and propidium monoazide (PMA) concentration were optimized to detect all viable pathogens in a single tube. A dual filtration system utilized a filtration membrane with different pore sizes to enrich pathogens found on fresh-cut cantaloupe. The results revealed that an optimized multiplex PCR system has the ability to effectively detect three pathogens in the same tube. The viable pathogens were simultaneously detected for PMA concentrations above 10 μg/ml. The combination of a nylon membrane (15 μm) and a micro pore filtration membrane (0.22 μm) formed the dual filtration system used to enrich pathogens. The achieved sensitivity of PMA-mPCR based on this dual filtration system was 2.6 × 10^3^ cfu/g for *L*. *monocytogenes*, 4.3 × 10 cfu/g for *E*. *coli* O157:H7, and 3.1 × 10^2^ cfu/g for *S*. *aureus*. Fresh-cut cantaloupe was inoculated with the three target pathogens using concentrations of 10^3^, 10^2^, 10, and 1 cfu/g. After 6-h of enrichment culture, assay sensitivity increased to 1 cfu/g for each of these pathogens. Thus, this technique represents an efficient and rapid detection tool for implementation on fresh-cut cantaloupe.

## Introduction

Cantaloupes (*Cucumis melo* L.) are an excellent source of vitamin A and C, as well as beta-carotene, potassium, dietary fiber, and iron [1]. The popularity of cantaloupes increases globally due to their high water content and low caloric value [2,3]. However, the relatively rough rind of cantaloupes may easily be contaminated with pathogens from irrigation water, soil, and fecal matter of animals [4–9]. Pathogens adhering to cantaloupe rinds can subsequently be translocated into the flesh during cantaloupe dressing [10,11]. During the past decade, twenty-nine foodborne disease outbreaks in the United States that were associated with cantaloupe affected 1751 consumers, of which 34 died due to the resulting infection [12]. Such outbreaks caused by the consumption of fresh produce have been associated with pathogens [13] and *E*. *coli* O157:H7 and *L*. *monocytogenes* infections have been predominantly associated with cantaloupes [14]. In 2011, an outbreak of *L*. *monocytogenes* due to cantaloupe contamination affected 146 consumers in 28 states, led to 32 deaths, and one miscarriage [12]. *S*. *aureus* and *E*. *coli* O157:H7 are able to survive and thrive on fresh-cut cantaloupes, although no cases of food poisoning have been associated with pathogens from cantaloupe [15]. Pathogen outbreaks and associated findings highlighted the significance for developing a highly specific, sensitive, and rapid detection technique to assure the food safety of fresh-cut cantaloupes.

Traditional detection methods first need to enrich the target pathogens, isolate bacterial pathogens from solid media, and confirm the infection and species via biochemical and serological tests. These procedures are extremely labor intensive and require significant time investment (form days to weeks) to yield a conclusive result. Multiplex polymerase chain reaction (mPCR) saves time and labor, and offers the advantage of simultaneous detection of different types of pathogenic bacteria [16–22]. However, the downside of this detection technology is that it cannot selectively distinguish between viable and dead bacteria [23]. DNA from dead bacterial cells can be amplified via mPCR. However, this technique shows several disadvantages including the necessity to eliminate any trace of the bacterial DNA that is present in the sample, limited sensitivity, reproducibility, and specificity [24]. Recently, the method of ethidium monoazide (EMA) or propidium monoazide (PMA) in combination with mPCR has been developed to enhance the accuracy of detection [25, 26]. The regent selectively penetrates only into the membrane-compromised structure of dead cells, where it intercalates into nucleic acids [27]. However, EMA has been reported to also penetrate into integral cell membranes and combined with genomic DNA during lighting, this results in the loss of partly viable cells [28, 29]. This demonstrated that the ability of PMA surpassed that of EMA in distinguishing between viable and dead cells of various bacterial species [30]. In addition, the PCR-base detection method for pathogens was affected by numerous factors, including acid-based fruit residue [19]. Microfiltration via different pore sizes is a rapid and simple procedure for filtering bacteria from mixed samples.

In this study, microfiltration-based multiplex PCR in combination with a PMA assay was developed for detection and discrimination of *E*. *coli* O157:H7, *L*. *monocytogenes*, and *S*. *aureus* on fresh-cut cantaloupes. Highly sensitive primers for specific pathogen genes were designed, resulting in an assay that can indeed detect all three viable pathogens simultaneously, even though cantaloupe debris can inhibit the PCR test. A microfiltration membrane was included to eliminate cantaloupe pulp interference, enhance pathogen enrichment and thus shorten detection time. The developed assay will represent a useful diagnostic tool during fresh-cut fruits processing, enabling the prevention of contaminated food distribution.

## Materials and Methods

### Bacterial strains

Bacterial strains used in this study are listed in Table 1. They were obtained from the China Center of Industrial Culture Collection (CICC, Beijing, China), the China General Microbiological Culture Collection Center (CGMCC, Beijing, China), the Guangdong Microbiology Culture Center (GIM, Guangdong, China), and the Microbiology safety laboratory of the Dalian Nationality University, China. *L*. *monocytogenes* was cultured in trypticase Soy Broth-Yeast Extract (TSB-YE), *S*. *aureus* was cultured in trypticase Soy Broth (TSB), and *E*. *coli* O157:H7 was cultured in Luria-Bertani (LB). Other bacterial strains were cultured in Nutrient Broth (NB). All pathogens were enumerated using *L*. *monocytogenes* chromogenic culture medium, *S*. *aureus* chromogenic culture medium, and *E*. *coli* O157:H7 chromogenic culture medium, respectively. All plates were incubated at 37°C for 24–48 h in order to enable adequate pathogen growth. All media were purchased from Qingdao Hope Bio-Technology Co., Ltd (Hopebio, Qingdao, China).

**Table 1:** List of all bacterial strains used in this study.

Bacteria strain (No.)	Source
*Listeria monocytogenes*	ATCC 19111
*Listeria monocytogenes*	ATCC 19112
*Listeria monocytogenes*	ATCC 19115
*Listeria monocytogenes*	ATCC 15313
*Listeria monocytogenes*	GIM 1.229
*Staphylococcus aureus*	ATCC 6538
*Staphylococcus aureus*	CICC 21600
*Staphylococcus aureus*	CICC 10201
*Staphylococcus aureus*	CICC 23656
*Escherichia coli* O157:H7	NCTC 12900
*Escherichia coli* O157:H7	CICC 21530
*Escherichia coli* O157:H7	CICC 10907
*Listeria ivanovii*	ATCC 19119
*Listeria grayi*	ATCC 25401
*Listeria seeligeri*	ATCC 35967
*Listeria welshimeri*	ATCC 35897
*Listeria innocua*	ATCC 33090
*Salmonella* Typhimurium	ATCC 14028
*Samonella enterica subsp*. *enterica*	CMCC 50115
*Salmonella paratyphi* Type B	CMCC 50094
*Salmonella enterica subsp*. *enterica*	CICC 10871
*Salmonella* Typhi	CMCC 50071
*Micrococcus luteus*	CMCC 28001
*Proteus mirabilis*	CMCC 49005
*Bacillus cereus*	CMCC 63301
*Escherichia coli*	CMCC 44102
*Escherichia coli* STEC	CICC 10668
*Escherichia coli* ETEC	CICC 10665
*Escherichia coli* ETEC O25: K19	CICC 10414
*Escherichia coli* EPEC O 127: K63	CICC 10411
*Escherichia coli* EIEC	CICC 10661
*Vibrio parahemolyticus*	CICC 21617
*Vibrio cholerae*	CICC 23794
*Enterobacter sakazakii*	CICC 21560
*Pseudomonas aeruginosa*	CICC 20236
*Campylobacter jejuni*	CICC 22936
*Shigella flexneri*	CICC 10865
*Shigella sonnei*	CICC 21679
*Pseudomonas fluorescens*	CICC 20225
*Yersinia enterocolitica*	CICC 10869
*Bacillus subtilis*	CICC 10275

### DNA extraction

Cell suspensions of all three pathogens were centrifuged at 15,000 × g for 2 min, respectively. Prior to DNA extraction, all samples were washed twice in an equal volume of sterile water. Genomic bacterial DNA was extracted using the MiniBEST Bacterial Genomic DNA Extraction kit Ver. 2.0 (Takara, Shiga, Japan). Concentration and purity of DNA templates were measured via Thermo Scientific Multiskan FC (Thermo Fisher Scientific, MA, USA). Subsequently, DNA templates with high purity within a ratio of 1.8–2.0 (A_260_ / A_280_) were diluted in distilled water to identical concentrations and then stored at -20°C.

### Primer design and screening

The *wzy* gene (GenBank: AF061251.1) of *E*. *coli* O157:H7, the *ina* gene (GenBank: EU295422.1) of *L*. *monocytogenes*, and the *nuc* gene (GenBank: DQ507379.1) of *S*. *aureus* were selected to design primers via Primer Premier 5.0 software (Premier Biosoft, CA, USA). Primer pairs were specifically designed and screened for this study to simultaneously detect three pathogens in a single reaction. The information of the three target genes, primer pairs, and amplicon sizes are listed in Table 2. Takara Bioengineering Co. (Dalian, China) synthesized the primers. Furthermore, specificity and verification of each primer pair was confirmed via single PCR using non-target bacterial strains.

**Table 2:** Primer pairs designed for the multiplex PCR in this study.

Microorganism	Primer sequence (5’-3’)	Length (bp)
*L*. *monocytogenes*	*ina*-F: GAGCTAACCAAATAAGTAACA	285
	*ina*-R: AGGTCGCTAATTTGGTTA	
*S*. *aureus*	*nuc*-F: TTCGCTACTAGTTGCTTA	159
	*nuc*-R: CGCAGGTTCTTTATGTAA	
*E*. *coli* O157:H7	*wzy*-F: GTTCCATATGTTGTTTCTGA	193
	*wzy*-R: CTGCTCCATACGTAGTAA	

### Multiplex PCR assay

Primer concentrations and reaction conditions were further optimized for multiplex PCR. The utilized reaction system contained 1.32 ng/μL, 1.40 ng/μL, and 1.25 ng/μL of extracted DNA as templates of *L*. *monocytogenes*, *S*. *aureus*, and *E*. *coli* O157:H7, respectively. The system furthermore contained, 5 μL of 10 × PCR buffer (Mg^2+^ plus), 0.20 mM of dNTPs, 0.08 μM each of forward and reverse *ina* primers, 0.1 μM each of forward and reverse *wzy* primers, 0.1 μM each of forward and reverse *nuc* primers, 0.5 U of Ex Taq DNA polymerase, and RNase-free water was used to replenish to a final volume of 50 μL. All regents for the PCR test were purchased from the Takara Bio company (Dalian, China).

The samples were amplified in an *ARKTIK* Thermal Cycler (Thermo Fisher Scientific, MA, USA). The mPCR reaction conditioning started with pre-denaturing with 95°C for 3 min, followed by 32 cycles of denaturing at 94°C for 40 s each, annealing temperature of 50°C for 30 s and an extension at 72°C for 40 s, followed by a final extension step at 72°C for 10 min. Subsequent to mPCR amplification, the products were subjected to 3% agarose gel including Gelred dye (Biotium, Inc., Hayward, CA, USA) for electrophoresis. The image was studied with the UVP BioSpectrum Imaging System (UVP, LLC, CA, USA).

### Internal verification and primer specificity

Multiplex PCR was performed on the mix of genomic DNA of the three pathogens (concentration for each at 10^8^ CFU/ml) using optimized conditions as described above. To test for the existence of disturbances among target pathogens, three DNA templates were amplified using two random primer pairs in a single reaction tube, using mPCR for the three pathogens as positive control. To investigate whether the presence of non-target bacteria disturbs identification and detection of target bacteria, non-target bacteria strains were detected via mPCR in the same assay. DNA templates were prepared as described above and immediately tested after isolation.

### Sensitivity test of the multiplex PCR

To determine the sensitivity of the mPCR assay, the pathogen cell suspension was serially diluted 10-fold with 0.1% peptone water after washing twice, resulting in concentrations from 10^8^ to 10^0^ CFU/mL. The suspensions were subjected to DNA extraction and were subsequently tested with the mPCR detection assay.

### Optimization of PMA treatment

*E*. *coli* O157:H7, *L*. *monocytogenes*, and *S*. *aureus* were cultured in LB, TSB-YE, and TSB media for 24 h. The suspensions were heat-treated at 100°C for 15 min and immediately immersed in ice for 2 min to obtain dead cells. Heat-treated cells and viable cells were confirmed onto LB plates, TSA media, and TSA-YE media.

PMA (Biotium, Inc., Hayward, CA, USA) was dissolved in 20% dimethyl sulfoxide and stored at -20°C in the dark. 5 μL, 10 μL, 20 μL, and 40 μL of PMA (1 mg/ml) were respectively added to 1 mL of bacterial suspension in a light-transparent 1.5 mL microcentrifuge tube. The tube was incubated in the dark at room temperature for 5 min, mixing every 30 s [31], to allow PMA to penetrate the dead cells and intercalate with the DNA. The sample was then placed on crushed ice at a distance of 15–20 cm from the light source and exposed to a 500-W halogen light source for 5 min [32]. The tube was shaken every 30 s to guarantee homogeneous light exposure. Genomic pathogen DNA (viable and dead) was extracted from PMA treated samples using the method described above. The sample was tested with the newly developed mPCR as described above.

### Optimizing the filtration membrane for fresh-cut cantaloupe

Whole cantaloupes were cleaned and cut into cubes of 1 cm × 1 cm × 1 cm using a sterile knife and cutting board in a sterile room. *L*. *monocytogenes*, *S*. *aureus*, and *E*. *coli* O157:H7 were inoculated on 10 g of fresh-cut cantaloupe at 10^8^ CFU/ml and then air dried for 1 h. Samples were mixed for 1 min using a homogenizer (Interscience, France) after adding 90 mL of 0.1% peptone water. Samples were removed using a filtration apparatus (Hangzhou Hengqing Technology Co., Ltd, China) with different types of filtration membranes (polypropylene membrane of 10, 20, and 40 μm; nylon membrane of 15, 40, and 60 μm). The homogeneous solution and pathogens were absorbed into sterile conical flasks using circulating water pumps (SHZ-II-type, Shanghai Eguiding Analytical Instrument Co., Ltd. China). The filtrate was diluted with 0.1% peptone water and enumerated via Chromogenic media. The filtrate was collected through 0.22 μm or 0.45 μm filtration membranes, respectively. The diagram was designed about this dual filtration system (S1 Fig). Filtration membranes were put into a centrifuge tube containing 10 ml of 0.1% peptone water and vibrated using a vortex mixer for 2 min. The mixture was enumerated using Chromogenic media (see above).

### Detection limit of pathogens in artificially contaminated fresh-cut cantaloupe

Cantaloupes were purchased from a local supermarket (Newmart, Dalian, China) to test the newly-developed filtration-based PMA-mPCR. Fresh-cut cantaloupes were tested for contamination with *L*. *monocytogenes*, *S*. *aureus*, and *E*. *coli* O157:H7 via conventional methods. Each sample was inoculated with 10^7^–1 cfu/g of *L*. *monocytogenes*, *S*. *aureus*, and *E*. *coli* O157:H7, respectively. The treated samples received additional 90 mL of 0.1% peptone water, followed by homogenization for 1 min with a homogenizer (Interscience, Saint Nom la Breteche, France). The suspension was microfiltered and PMA treated, before the genomic pathogen DNA was extracted using the method above and amplified with mPCR. Each sample treatment was performed in triplicate.

### Incubation enrichment

Each sample (10 g) was inoculated with 1 mL of *L*. *monocytogenes*, *S*. *aureus*, and *E*. *coli* O157:H7 with 10^3^–1 cfu/g, respectively. Treated samples were topped up to 90 mL TSB for culture. Incubation durations were 0 h, 3 h, and 6 h and samples were homogenized for 1 min with a homogenizer. The suspension was then microfiltered and PMA treated, pathogen colonies were counted, while genomic DNA of pathogens was simultaneously extracted using the method described above, then amplified via multiplex PCR. Each treatment sample was performed in triplicate.

## Results

### Optimization of multiplex PCR

**Fig 1:**
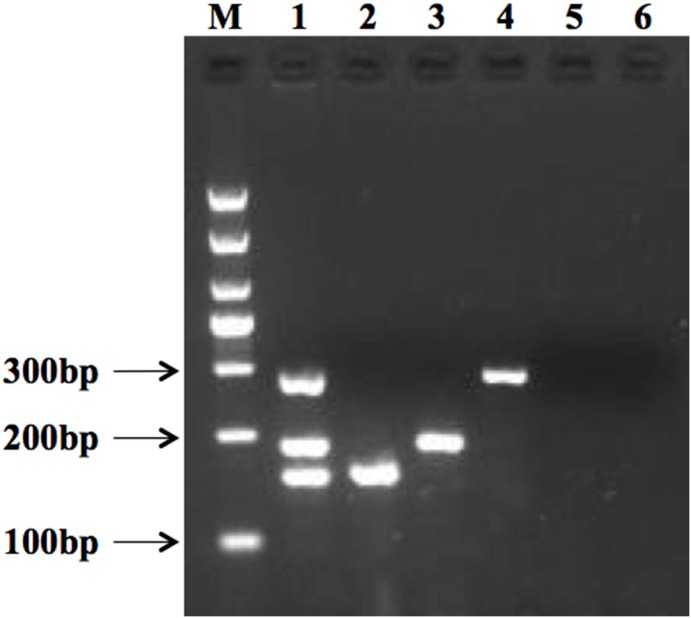
Multiplex PCR result of *L*. *monocytogenes*, *E*. *coli* O157:H7, and *S*. *aureus*, respectively. M: 1000 bp DNA marker. Lane 1 shows PCR amplicons, specific to *L*. *monocytogenes* (285 bp), *E*. *coli* O157:H7 (193 bp), and *S*. *aureus* (159 bp). Lanes 2–4 show individual PCR amplicons, specific to *S*. *aureus* (159 bp) (lane 2), *E*. *coli* O157:H7 (193 bp) (lane 3), and *L*. *monocytogenes* (285 bp) (lane 4). Lanes 5–6 show results for the negative control.

Reaction condition, annealing temperature, extending time, primer concentration, dNTP, and enzyme activity of mPCR were optimized to obtain three similar bands in a single tube, while preventing non-specific reactions in the control sample. Fig 1 shows the results of gel electrophoresis comparing the mPCR method that was established for three pathogens with specific PCR reactions for each pathogen individually. As control, the amplification result of ddH_2_O used the three pairs of primers that were studied in this paper. The target genes specific to *L*. *monocytogenes*, *S*. *aureus*, and *E*. *coli* O157:H7 produced amplicons at 285 bp, 159 bp, and 193 bp, respectively. This revealed distinct and bright bands at each band. No amplification band was found at the negative control.

### Primer specificity

**Fig 2:**
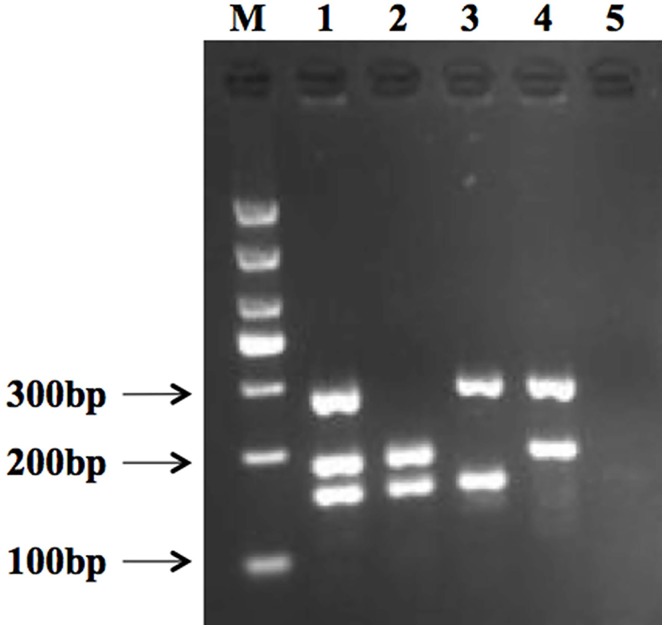
Internal verification and specificity of primers for *L*. *monocytogenes*, *E*. *coli* O157:H7, and *S*. *aureus*. Lane M: 1000 bp DNA marker. Lane 1 shows PCR amplicons specific to *L*. *monocytogenes* (285 bp), *E*. *coli* O157:H7 (193 bp), and *S*. *aureus* (159 bp). Lane 2 shows PCR amplicons specific to *S*. *aureus* (159 bp) and *E*. *coli* O157:H7 (193 bp). Lane 3 shows PCR amplicons specific to *L*. *monocytogenes* (285 bp) and *S*. *aureus* (159 bp). Lane 4 shows PCR amplicons specific to *E*. *coli* O157:H7 (193 bp) and *L*. *monocytogenes* (285 bp). Lane 5 shows results for the negative control.

**Fig 3:**
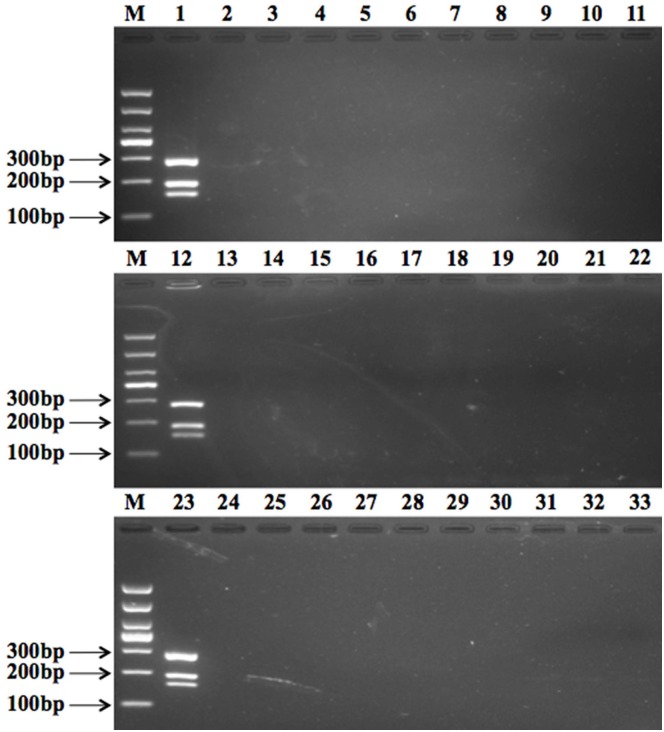
Primer specificity for *L*. *monocytogenes*, *E*. *coli* O157:H7, and *S*. *aureus*. Lane M: 2000 bp DNA marker; Lanes 1, 12, and 23 show the PCR amplicons specific to *L*. *monocytogenes* (285 bp), *E*. *coli* O157:H7 (193 bp), and *S*. *aureus* (159 bp); Lanes 2–11 show PCR amplicons specific to *Listeria ivanovii* (ATCC 19119), *Listeria grayi* (ATCC 25401), *Listeria seeligeri* (ATCC 35967), *Listeria welshimeri* (ATCC 35897), *Listeria innocua* (ATCC 33090), *Salmonella* Typhimurium (ATCC 14028), *Samonella enterica subspenterica* (CMCC 50115), *Salmonella paratyphi* Type B (CMCC 50094), *Salmonella enterica subsp*. *enterica* (CICC 10871), and *Salmonella* Typhi (CMCC 50071); Lanes 12–22 show PCR amplicons specific to *Micrococcus luteus* (CMCC 28001), *Proteus mirabilis* (CMCC 49005), *Bacillus cereus* (CMCC 63301), *Escherichia coli* (CMCC 44102), *Escherichia coli* STEC (CICC10668), *Escherichia coli* ETEC (CICC10665), *Escherichia coli* ETEC O25:K19 (CICC 10414), *Escherichia coli* EPEC O127:K63 (CICC 10411), *Escherichia coli* EIEC (CICC 10661), and *Vibrio parahemolyticus* (CICC 21617); Lanes 23–32 show PCR amplicons specific to *Vibrio cholera* (CICC 23794), *Enterobacter sakazakii* (CICC 21560), *Pseudomonas aeruginosa* (CICC 20236), *Campylobacter jejuni* (CICC 22936), *Shigella flexneri* (CICC 10865), *Shigella sonnei* (CICC 21679), *Pseudomonas fluorescens* (CICC 20225), *Yersinia enterocolitica* (CICC 10869), and *Bacillus subtilis* (CICC 10275); Lane 33 is the negative control.

Efficiency and specificity of the primers were assayed with target and non-target strains. Under optimized multiplex PCR conditions, a DNA mixture of three pathogens produced three bands (Fig 2, lane 1), which included *L*. *monocytogenes* (285 bp), *S*. *aureus* (159 bp), and *E*. *coli* O157:H7 (193 bp). Lanes 2 to 4 show two bands containing two random pathogens, each. Fig 2, lane 5 shows the result of the negative control. A total of 41 type strains including 29 non-target strains were evaluated via this multiplex PCR assay to confirm the specificity of three primer pairs (Fig 2). The result demonstrates that *L*. *monocytogenes*, *S*. *aureus*, and *E*. *coli* O157:H7 are amplified effectively and that no target pathogen produced a negative result (Fig 3).

### The sensitivity of the developed multiplex PCR

**Fig 4:**
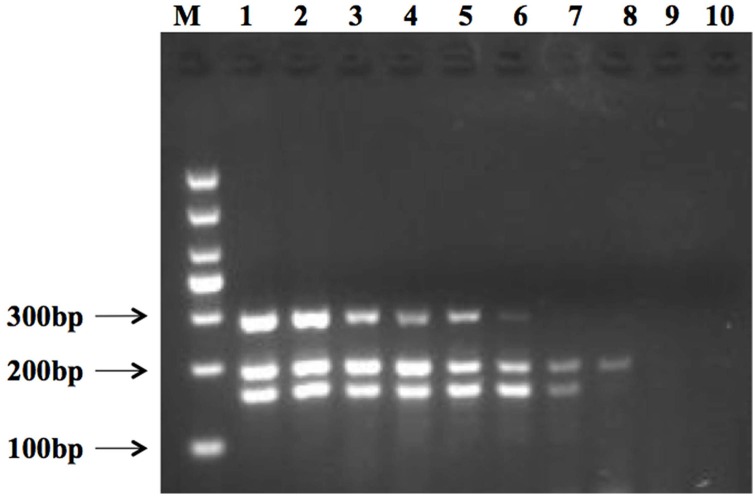
The sensitivity of the multiplex PCR assay using 10-fold serial dilutions of *L*. *monocytogenes*, *E*. *coli* O157:H7, and *S*. *aureus*. Lanes 1 to 8 show amplicon results for *L*. *monocytogenes* (from 2.3 × 10^8^ cfu/ml to 2.3 × 10 cfu/ml), *E*. *coli* O157:H7 (from 1.8 × 10^8^ cfu/ml to 1.8 × 10 cfu/ml), and *S*. *aureus* (from 3.4 × 10^8^ cfu/ml to 3.4 × 10 cfu/ml). Lane 9 and Lane 10 show the results of the negative controls.

To investigate the sensitivity of this optimized multiplex PCR, the genomic DNA of three pathogens was mixed (containing 10^8^ cfu/ml), serially diluting the suspension 10-fold to a concentration of 10 cfu/ml (Fig 4). The result revealed a sensitivity of the multiplex PCR for mixed genomic DNA of *L*. *monocytogenes* (2.3 × 10^3^ cfu/ml), *E*. *coli* O157:H7 (1.8 × 10^3^ cfu/ml), and *S*. *aureus* (3.4 × 10^3^ cfu/ml). The sensitivity of *L*. *monocytogenes* was 2.3 × 10^3^ cfu/ml, that of *E*. *coli* O157:H7 was 1.8 × 10 cfu/ml, and that of *S*. *aureus* was 3.4 × 10^2^ cfu/ml. All experiments were performed in triplicate.

### Concentration optimization with PMA treatment

**Fig 5:**
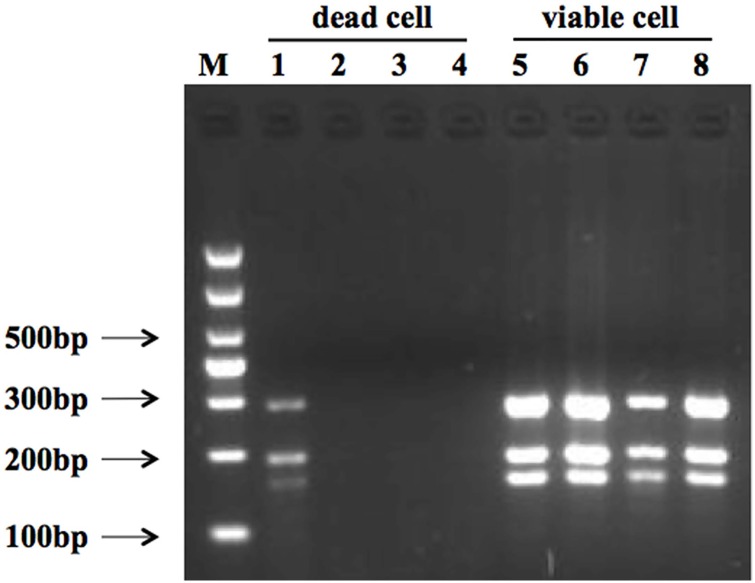
Result of multiplex PCR detection for dead and viable pathogens via PMA treatment. Lane M shows the 1000 bp DNA marker. Lanes 1–4 show genomic DNA extracted from dead *L*. *monocytogenes*, *E*. *coli* O157:H7, and *S*. *aureus* following PMA treatment (5 μg/ml, 10 μg/ml, 20 μg/ml, and 40 μg/ml, respectively). Lanes 5–8 show genomic DNA extracted from viable *L*. *monocytogenes*, *E*. *coli* O157:H7, and *S*. *aureus* after PMA treatment (5 μg/ml, 10 μg/ml, 20 μg/ml, and 40 μg/ml).

Following the development of the mPCR assay for *L*. *monocytogenes*, *S*. *aureus*, and *E*. *coli* O157:H7, the concentrations of the PMA treatment were optimized. Whole cells of all pathogens were heat-killed prior to PMA treatment, resulting in a colony count of zero on corresponding media. Dead cells treated with 5 μg/ml PMA resulted in the indistinct PCR signal of lane 2 of Fig 5. No target genes were detected on dead cells treated with PMA of concentrations of 10, 20, or 40 μg/ml. However, treating viable cells with PMA of different concentrations (5, 10, 20, or 40 μg/ml) resulted in three distinct PCR signals (Fig 5, lanes 6–9). The intensity of the band revealed a weakening tendency for viable cells for PMA concentrations of 20 and 40 μg/ml. A PMA treatment with 10 μg/ml was chosen, considering both the result for viable cells and economic cost.

### Optimizing the filtration membrane for fresh-cut cantaloupe

Different types of filtration membranes were chosen (polypropylene and nylon membranes) to enhance detection limit and reduce detection time. The initial inoculation level was respectively 8.90 log cfu/ml of *L*. *monocytogenes*, 8.70 log cfu/ml of *E*. *coli* O157:H7, and 8.38 log cfu/ml of *S*. *aureus*. The enrichment and filtration result of target pathogen based on polypropylene membrane was approximate 10^7^ cfu/ml (Table 3). Table 4 lists the colony count using a nylon membrane filtration, representing a filtration level of 10^8^ cfu/ml. This result was more similar to the number of inoculations level than that on polypropylene membrane. Nylon membranes with three different pore sizes (15 μm, 40 μm, and 60 μm) all demonstrated a similar ability to elute bacteria. The nylon membrane with 15 μm pore size was chosen for cantaloupe residue removal, considering that pore sizes of 40 μm and 60 μm would allow more residue to pass through during filtration. The capacity to enrich bacteria using a filtration membrane of 0.22 μm pore size was superior to that of a 0.45 μm filtration membrane. Thus, a re-filtration membrane with pore size of 0.22 μm was chosen to enrich bacteria using identical filtration equipment.

**Table 3:** Number of bacterial colonies for *Listeria monocytogenes*, *Escherichia coli* O157:H7, and *Staphylococcus aureus* inoculated on fresh-cut cantaloupe based on Polypropylene membrane.

Filtration (log cfu/ml)	Polypropylene membrane (log cfu/ml)					
	10 μM		20 μM		40 μM	
Re-filtration	0.22 μM	0.45 μM	0.22 μM	0.45 μM	0.22 μM	0.45 μM
*L*. *monocytogenes* (8.90±0.03)	7.19±0.06	7.09±0.07	7.44±0.03	7.22±0.04	7.82±0.02	7.64±0.03
*E*. *coli* O157:H7 (8.70±0.02)	7.57±0.01	7.18±0.08	7.71±0.02	7.50±0.03	7.84±0.02	7.62±0.03
*S*. *aureus* (8.38±0.03)	7.69±0.04	7.39±0.04	7.79±0.02	7.56±0.03	7.98±0.01	7.73±0.03

**Table 4:** Number of bacterial colonies for *Listeria monocytogenes*, *Escherichia coli* O157:H7, and *Staphylococcus aureus* inoculated on fresh-cut cantaloupe based on nylon membrane.

Filtration (log cfu/ml)	Nylon membrane (log cfu/ml)					
	15 μM		40 μM		60 μM	
Re-filtration	0.22 μM	0.45 μM	0.22 μM	0.45 μM	0.22 μM	0.45 μM
*L*. *monocytogenes* (8.90±0.03)	8.59±0.04	8.19±0.09	8.60±0.05	8.48±0.09	8.64±0.04	8.11±0.12
*E*. *coli* O157:H7 (8.70±0.02)	8.15±0.13	7.97±0.03	8.26±0.13	7.94±0.03	8.35±0.08	8.16±0.16
*S*. *aureus* (8.38±0.03)	8.06±0.09	7.95±0.03	8.10±0.12	8.10±0.11	8.25±0.09	7.98±0.04

### Detection limit of viable pathogens on artificially contaminated fresh-cut cantaloupe

**Fig 6:**
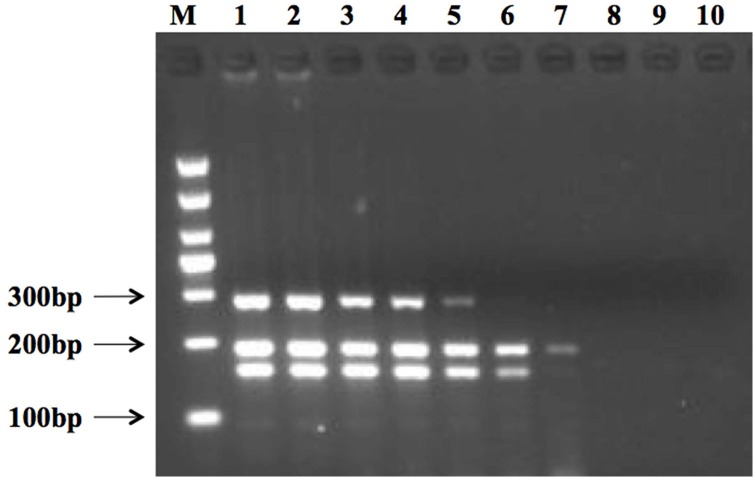
Detection limit of the multiplex PCR assay using 10-fold serial dilutions of populations of *L*. *monocytogenes*, *E*. *coli* O157:H7, and *S*. *aureus* on fresh-cut cantaloupe. Lane M shows the 1000 bp DNA marker. Lanes 1 to 8 show amplicon results for *L*. *monocytogenes* (from 2.6 × 10^7^ cfu/g to 1 cfu/g), *E*. *coli* O157:H7 (from 4.3 × 10^7^ cfu/g to 1 cfu/g), and *S*. *aureus* (from 3.1 × 10^7^ cfu/g to 1 cfu/g). Lanes 9 and 10 show results for the negative control.

The mPCR sensitivity test was further investigated using fresh-cut cantaloupe that had been inoculated with different concentrations of the three pathogens (10^7^–1 cfu/g). DNA was directly extracted via PMA treatment from the sample subsequent to filtration and without prior enrichment step. The mPCR result revealed a detection limit for a combination of *L*. *monocytogenes* of 2.6 × 10^3^ cfu/g, *E*. *coli* O157:H7 of 4.3 × 10^3^ cfu/g, and *S*. *aureus* of 3.1 × 10^3^ cfu/g, while for *L*. *monocytogenes* alone, the detection limit was 2.6 × 10^3^ cfu/g, for *E*. *coli* O157:H7 alone, it was 4.3 × 10 cfu/g, and for *S*. *aureus* alone, the detection limit was 3.1 × 10^2^ cfu/g (Fig 6).

### Pathogen enrichment on artificially contaminated fresh-cut cantaloupe

**Fig 7:**
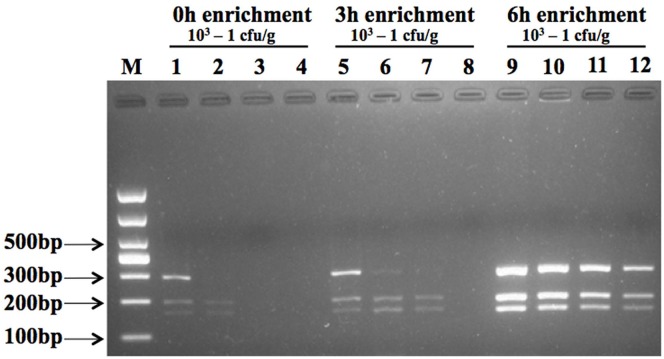
Detection of multiplex PCR assay on fresh-cut cantaloupe inoculated with 10^3^–1 cfu/g following prior enrichment. Lane M shows 1000 bp DNA marker. Lanes 1 to 4, lanes 5 to 8, and lanes 9 to 12 show amplicon results after 0, 3, 6 h enrichment, respectively for *L*. *monocytogenes*, *E*. *coli* O157:H7, and *S*. *aureus* on fresh-cut cantaloupe that had been inoculated with 1.8 × 10^3^–1 cfu/g, 1.1 × 10^3^–1 cfu/g, and 1.4 × 10^3^–1 cfu/g.

Three pathogens were used for multiplex PCR amplification at different levels of inoculation (1.8 × 10^3^–1 cfu/g for *L*. *monocytogenes*, 1.1 × 10^3^–1 cfu/g for *E*. *coli* O157:H7, and 1.4 × 10^3^–1 cfu/g for *S*. *aureus*) either immediately (Fig 7, lanes 1–4), after 3 h (Fig 7, lanes 5–8), or after 6 h (Fig 7, lanes 9–12) of enrichment culture. The result revealed three evident, but hazy target bands from all three pathogens for an inoculation level of 10^3^ cfu/g immediately subsequent to enrichment (Fig 7, lane 1). DNA from *S*. *aureus* and *E*. *coli* O157:H7 could be amplified for an inoculation level of 10^2^ cfu/g (Fig 7, lane 2). No target band was obtained with an inoculation level of 10 cfu/g and 1 cfu/g without prior enrichment (Fig 7, lane 3–4). The result revealed three hazy target bands when the initial level of inoculation was 10^2^ cfu/g after 3 h of enrichment (Fig 7, lane 6). The band representing DNA of *L*. *monocytogenes* was faint and minor compared to other bands. No single band was amplified with this multiplex PCR method with an initial inoculation level of 1 cfu/g after 3 h of enrichment (Fig 7, lane 8). Three target bands were effectively amplified for initial inoculation levels from 10^3^ to 1 cfu/g following 6 h enrichment (Fig 7, lane 9–12). This demonstrates that this multiplex PCR detection method worked under inoculation levels as low as 1 cfu/g with prior enrichment of 6 h. The colony number of target pathogen was enumerated after corresponding enrichment time (0, 3h, 6h) (S1 Fig)

## Discussion

Many studies of *L*. *monocytogenes*, *S*. *aureus*, and *E*. *coli* O157:H7 food sample infection incidences have been reported. These studies included pathogen contaminations of cooked foods [33], raw vegetables [34], and broilers [35,36]. In addition, outbreaks of pathogen contamination in minimally processed fruits from street merchants were significantly higher compared to those from supermarkets [37].

Culture-based detection methods have been widely used so far; however, they pose severe limitations, such as long detection duration, false negatives, and high labor-intensity. Numerous methods of molecular biology have been developed to detect pathogenic bacteria in food [38,39]. Prominent examples are PCR [40], real time PCR [41,42], loop-mediated isothermal amplification (LAMP), and DNA microarray analysis [43,44]. However, multiplex PCR with simultaneous detection of more that one pathogen, low cost, and low labor intensity is far more convenient and quicker than other methods of molecular biology.

To our knowledge, this is the first report that uses a filtration-based PMA-mPCR detection method to distinguish between dead and viable *L*. *monocytogenes*, *S*. *aureus*, and *E*. *coli* O157:H7 on fresh-cut cantaloupe. Compared to conventional biochemical tests that require more labor, material, and time, the method presented in this study has definite advantages [45]. In order to establish this mPCR system, three primers were designed to specifically amplify different sizes of target genes. Three primer pairs were used to simultaneously ensure identification of *L*. *monocytogenes*, *S*. *aureus*, and *E*. *coli* O157:H7 within the same reaction tube and even in the presence of other related and non-related bacterial strains.

Such mPCR assays using specific primers have been indicated to be efficient for the detection of pathogenic bacteria on food products [46]. However, a DNA-based detection method cannot discriminate between viable and dead cells. The number of viable cells on food samples has been overestimated due to false positives [47]. To remove the effects introduced by dead cells within the PCR signals, EMA, PMA, and Reagent D treatment was applied to crosslink with the DNA of dead cells [48].

In this study, PMA was selected due to superior selectivity in penetrating dead cells, while EMA and Reagent D have also been confirmed to penetrate membranes of viable cells [49–51]. A PMA concentration optimization revealed that a PMA concentration of 40 μg/mL did not inhibit the amplification of target DNA from the viable three pathogens. This was higher than the 20 μg/mL reported for *E*. *coli* [52]. However, the minimum PMA concentration that completely inhibited DNA amplification from dead cells was 10 μg/mL, notably higher than the 3 μg/mL previously reported for *E*. *coli* [52]. The indistinct band in lane 1 of Fig 5 revealed that the test could not distinguish between viable and dead cells for a PMA concentration of 5 μg/mL. A further study reported that false positive results still occured for the quantitative detection of *Vibrio parahaemolyticus* for a PMA concentration below 8 μg/ml [32]. Based on the obtained result, a final PMA concentration of 10 μg/mL resulted in complete elimination of PCR signals from dead target bacteria cells, without PCR signal reduction from viable cells.

However, factors such as inhibition and residue of samples can affect direct detection of pathogens on contaminated samples via this PCR system. To circumvent this problem, methods were developed, such as microfiltration [53], IMS [21], and filtration [54]. In this study, filtration system-based methods proved effective in removing residue and inhibition from fresh-cut cantaloupe. A filtration membrane pore size beyond 10 μm allows most pathogens to pass. However, large particles such as fruit pulp were blocked from passing the filtration membrane. Most of the viable bacteria can be recovered on the surface of both 0.22 and 0.45 μm filtration membranes. This agrees with published data on pore sizes enabling different bacterial species to pass through a filtration membrane [54]. The passing ability of pathogens strongly depends on the type of filtration membrane. Polycarbonate screen membranes enabled cells to pass more effectively and had a very distinct threshold at which no further cells would pass the membrane. Cellulose ester membranes showed a more gradual reduction of cell numbers in the filtrate as pore size decreased and cells ceased to pass through the membranes [55].

Moreover, the sensitivity of the multiplex PCR assay described in this study was in agreement with a previous report by Patricia and Rosa [56]. The authors tested a multiplex PCR assay on artificially inoculated fresh and minimally processed vegetables, revealing a sensitivity of 10^3^ CFU/g for direct detection of *E*. *coli* O157:H7, *Salmonella* spp., and *S*. *aureus*. Similarly, the report revealed that a detection limiting of 10^4^ CFU/ml could be achieved for *L*. *monocytogens*, *S*. *aureus*, *Strep*. *agalactiae*, *Ent*. *sakazakii*, *E*. *coli* O157:H7, *V*. *parahaemolyticus*, *Salmonella* spp., and *P*. *fluorescens* without relying on a prior enrichment step via multiplex PCR [57]. However, the detection limits of viable *E*. *coli* O157:H7 and *S*. *aureus* in this study were lower than in both of these reports. Several studies have confirmed that the sensitivity of multiplex PCR for the detection of pathogens can be further enhanced after enrichment. Ferretti et al. [58] have obtained excellent results using a 12-h enrichment and PCR-based test for *Salmonella* spp. in naturally contaminated salami. The report revealed a detection limit of 10^3^ cfu/ml for *Campylobacter* spp. and 10^6^ cfu/ml for *Salmonella* spp. of spiked chicken meat rinse without a prior enrichment step. Following 24-h enrichment, assay sensitivity was increased and detected up to 1 cfu/ml in samples contaminated with 1−10^5^ cfu/ml via multiplex real-time PCR for both pathogens [59]. *E*. *coli* O157:H7, *Salmonella enterica*, and *L*. *monocytogenes* were inoculated at 10 cfu/g. Similarly, samples of spinach, egg, and hotdog were inoculated with 10 CFU/g of *E*. *coli* O157:H7, *Salmonella* enterica, and *L*. *monocytogenes*, respectively could be detected via quantitative PCR (qPCR) after an enrichment period of 7 h [60]. This demonstrated that enrichment is a helpful step to remove inhibitors, improve the recovery of pathogens, and enhance the reaction stability for detection. In this study, three pathogens could be amplified via multiplex PCR under different inoculation levels after 6 h of enrichment culture. The achieved detection sensitivity was 1 cfu/g. This result was consistent with other reports; however, the shorter necessary enrichment time of 6 h reveals the superiority of the detection method introduced in this study.

## Conclusions

In conclusion, the novel filtration-base PMA-mPCR assay designed for this study was sensitive, swift, and specific for the simultaneous detection of viable *L*. *monocytogenes*, *S*. *aureus*, and *E*. *coli* O157:H7 on fresh-cut cantaloupe. The combination of the PMA-mPCR assay with the appropriate filtration membrane effectively eliminated the inhibitory effect of food samples. With this method, target pathogens can be detected after 6 h in a single reaction, reducing time and labor costs. In addition, the rapid detection of these pathogens, allows food supply monitors to immediately take appropriate measures to prevent the distribution of contaminated food. Thus, this assay promises to be an efficient diagnostic tool for the implementation on fresh-cut cantaloupe. This study revealed the combination of PMA-multiplex PCR and the filtration membrane method increased the detection success of *L*. *monocytogenes*, *S*. *aureus*, and *E*. *coli* O157:H7.

## Supporting Information

S1 Fig The diagram of dual filtration system. (TIF)

S1 Table The colony number of target pathogen after enrichment 0, 3, 6h. (XLSX)
