# Supplementary figures and images for: A Dual Filtration-Based Multiplex PCR Method for Simultaneous Detection of Viable Escherichia coli O157:H7, Listeria monocytogenes, and Staphylococcus aureus on Fresh-Cut Cantaloupe
Source: PLoS One. 2016 Dec 1;11(12):e0166874. doi: 10.1371/journal.pone.0166874 (PMC5132219; doi:10.1371/journal.pone.0166874)

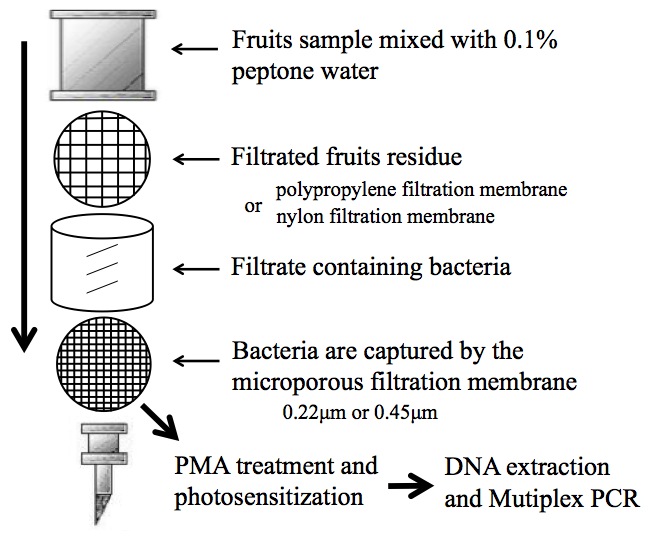

Supplement: S1 Fig — (TIF) [file pone.0166874.s001.tif]
